# Identification of *Leishmania major* UDP-Sugar Pyrophosphorylase Inhibitors Using Biosensor-Based Small Molecule Fragment Library Screening

**DOI:** 10.3390/molecules24050996

**Published:** 2019-03-12

**Authors:** Ohm Prakash, Jana Führing, John Post, Sharon M. Shepherd, Thomas C. Eadsforth, David Gray, Roman Fedorov, Françoise H. Routier

**Affiliations:** 1 Department of Clinical Biochemistry OE4340, Hannover Medical School, Carl-Neuberg-Strasse 1, 30625 Hannover, Germany; Prakash.Ohm@mh-hannover.de (O.P.); Fuehring.Jana@mh-hannover.de (J.F.); 2 School of Life Sciences, University of Dundee, Dow Street, Dundee DD1 5EH, UK; j.m.z.post@dundee.ac.uk (J.P.); S.M.Shepherd@dundee.ac.uk (S.M.S.); t.eadsforth@dundee.ac.uk (T.C.E.); d.w.gray@dundee.ac.uk (D.G.); 3 Institute for Biophysical Chemistry, Hannover Medical School, Carl-Neuberg-Strasse 1, 30625 Hannover, Germany; Fedorov.Roman@mh-hannover.de

**Keywords:** UDP-sugar pyrophosphorylase, allosteric inhibitor, inhibitor scaffold, library screen

## Abstract

Leishmaniasis is a neglected disease that is caused by different species of the protozoan parasite *Leishmania*, and it currently affects 12 million people worldwide. The antileishmanial therapeutic arsenal remains very limited in number and efficacy, and there is no vaccine for this parasitic disease. One pathway that has been genetically validated as an antileishmanial drug target is the biosynthesis of uridine diphosphate-glucose (UDP-Glc), and its direct derivative UDP-galactose (UDP-Gal). De novo biosynthesis of these two nucleotide sugars is controlled by the specific UDP-glucose pyrophosphorylase (UGP). *Leishmania* parasites additionally express a UDP-sugar pyrophosphorylase (USP) responsible for monosaccharides salvage that is able to generate both UDP-Gal and UDP-Glc. The inactivation of the two parasite pyrophosphorylases UGP and USP, results in parasite death. The present study reports on the identification of structurally diverse scaffolds for the development of USP inhibitors by fragment library screening. Based on this screening, we selected a small set of commercially available compounds, and identified molecules that inhibit both *Leishmania major* USP and UGP, with a half-maximal inhibitory concentration in the 100 µM range. The inhibitors were predicted to bind at allosteric regulation sites, which were validated by mutagenesis studies. This study sets the stage for the development of potent USP inhibitors.

## 1. Introduction

Leishmaniasis, categorized as a "Neglected Tropical Disease by WHO, affects about 12 million people worldwide and is often related to poverty [1]. The causative agents of this disease are protozoan flagellates of the genus *Leishmania*, and there are over 20 different species that are reported to cause this disease in humans, as well as in various synanthropic animal hosts [2]. Depending on the infecting *Leishmania* species and the immune status of the host, clinical manifestation can vary from disfiguring cutaneous or mucocutaneous forms, to a life-threatening visceral form [3,4]. The mortality due to visceral leishmaniasis has decreased from approximately 62,000 to 14,000 casualties between 2013 and 2016 [5,6]. However, leishmaniasis remains associated with high morbidity. As of now, no vaccines are available against this disease [7]. Growing concern over reports of drug resistance and adverse effects due to the overuse of available drugs calls for the discovery of cost- and therapeutically effective, parasite-specific lead compounds against this disease [8,9].

Leishmaniasis is spread through the bite of a female Phlebotomine sandfly, the insect vector of the parasite. Within the intestinal tract of the insect vector, *Leishmania* exists as an extracellular flagellated promastigote, whereas in mammalian hosts, it is predominantly found intracellularly in the macrophage phagolysosomes as a flagellated amastigote [10]. In order to adapt to the changing environmental conditions during its digenetic life cycle, *Leishmania* undergoes various morphological, physiological, and biochemical changes. The most prominent one is the synthesis of a dense surface glycocalyx, which contains various GPI-anchored molecules, including glycoproteins, lipophosphoglycans (LPGs), proteophosphoglycans (PPGs), and abundant glycoinositolphospholipids (GIPLs). Leishmania also secretes various glycoconjugates, especially proteophosphoglycans (PPGs) [11]. The secreted and surface glycoconjugates of the promastigote play a role in survival, infectivity, or virulence but are dispensable for *Leishmania* viability in culture [11,12].

For the biosynthesis of glycoconjugates, *Leishmania* parasites require an abundant supply of nucleotide sugars. They synthesize uridine diphosphate-glucose (UDP-Glc), UDP-galactose (UDP-Gal), UDP-*N*-acetylglucosamine (UDP-GlcNAc), UDP-galactofuranose (UDP-Gal*f*), GDP-mannose (GDP-Man), and GDP-arabinose (GDP-Ara), of which UDP-Glc has the highest steady-state pool size [13]. In addition to its contribution towards glycocalyx biosynthesis, UDP-Glc is involved in base J (β-d-glucosyl-hydroxymethyluracil) biosynthesis, an unusual DNA base that is found in *Leishmania* parasites [14,15]. It is assumed that base J plays an important role in parasite growth since attempts to delete JBP1, a gene encoding one of the two thymidine hydroxylases involved in the first step of base J synthesis, were unsuccessful [15,16]. Base J occurs in telomeric repeats and at transcription initiation and termination sites, and plays a role in transcription regulation [17]. Finally, UDP-Glc is the major source for glucosylation of various glycoproteins in the endoplasmic reticulum mediated by the enzyme UDP-glucose: glycoprotein glucosyltransferase (UGGT) which is known to be involved in the protein folding machinery [18]. In the related parasite *Trypanosoma brucei*, UGGT is not essential for parasite growth under standard culture conditions [19].

In prokaryotes and eukaryotes, the de novo synthesis of UDP-Glc is catalyzed by a specific UDP-glucose pyrophosphorylase (UGP) from the substrates uridine triphosphate (UTP) and glucose-1-phosphate (Glc-1P). UDP-Glc is then partially converted into UDP-Gal by the UDP-Glc 4′-epimerase [20]. In addition to this de novo pathway, *Leishmania* parasites and plants possess a UDP-sugar pyrophosphorylase (USP), which has broad substrate specificity and is involved in monosaccharide salvage [21,22,23,24]. In vitro, *Leishmania* and plant USPs preferentially use UTP as a nucleotide donor, and can activate a variety of sugar-1-phosphate [21,25]. These enzymes are most active with galactose-1-phosphate (Gal-1P) and Glc-1P, with a slight preference for the former, but they cannot activate *N*-acetylated sugars [21,25]. In contrast, UGP is highly specific for Glc-1P. UDP-sugar-producing pyrophosphorylases catalyze reversible reactions, require magnesium for catalysis, and act through an ordered sequential Bi-Bi reaction mechanism. In the forward reaction, UTP binds before the sugar-1P, and after catalysis, inorganic pyrophosphate (PPi) is released before the UDP-sugar [21,26,27]. The crystal structures of *Leishmania major* USP [28] and UGPs from several eukaryotic organisms have been elucidated [29,30,31,32,33,34] and they reveal a common tripartite structure with a small N-terminal domain, a large central catalytic domain with a Rossmann-like fold, and a β-helix C-terminal domain. An important difference between parasite (and plants) pyrophosphorylases and human UGP lies in their oligomerization states. Both *Leishmania* UGP and USP are active in their respective monomeric forms, whereas human UGP (*h*UGP) is an active octamer [21,26,35]. In the monomeric enzymes, the stepwise binding of the substrates causes vast structural rearrangement, leading first to the formation of the sugar-1P binding pocket, and then to intramolecular closure of the active site. In contrast, the human enzyme uses an intermolecular mechanism, in which interactions between the subunits stabilizes the sugar binding region [34].

Deletion of the UGP-encoding gene in *L. major* strongly reduced, but did not abolish the biosynthesis of UDP-Gal and its immediate precursor UDP-Glc, since USP enables the limited biosynthesis of both nucleotide sugars [36]. Deletion of one of the two *usp* alleles in a UGP-deficient parasite strain (*ugp*^−/−^), and conditional degradation of the enzyme produced by the remaining *usp* allele led to parasite growth arrest and cell death. Thus, the biosynthesis of UDP-Gal and/or UDP-Glc is essential for *L. major* survival [37]. Biosynthesis of UDP-Gal is also essential for the trypanosomatid parasites *Trypanosoma brucei* [38,39] and *Trypanosoma cruzi* [40]. However, in these two parasites, the only pathway for UDP-Gal biosynthesis is via UGP and the UDP-Glc 4′-epimerase. Although *T. cruzi* genomes encode a USP, the hexose transporter of this parasite does not import galactose into the cell [41]. Thus potent and specific inhibitors of the enzymes involved in UDP-Glc/UDP-Gal biosynthesis may represent novel therapeutics to combat infections that are caused by trypanosomatid parasites.

Enzymes of the uridylyltransferase family across all species exhibit highly conserved active sites at primary, secondary, and tertiary structural levels. Thereby, inhibitors targeting the catalytic sites of the enzymes would likely show cross-reactivity and off-target effects. An approach to achieve selective inhibition would employ allosteric regulation sites, which often have a unique composition and conformational properties [42,43]. In previous work, murrayamine-I was identified as an allosteric inhibitor of *L. major* UGP [42]. However, the suppression of the UDP-Glc/UDP-Gal biosynthesis in *Leishmania* parasites would require the inhibition of both UGP and USP. In this study, we therefore looked for specific inhibitors of *Leishmania* USP through a combination of biochemical and computational approaches, and demonstrated the possibility of developing a compound that is able to selectively inhibit both *Leishmania* USP and UGP.

## 2. Results

### 2.1. Identification of Scaffolds for Inhibitor Development by Small-Molecule Fragment Screening

To identify scaffolds for the development of *Leishmania* USP inhibitors, a chemical library of 965 fragment molecules from the University of Dundee Drug Discovery Unit (DDU) was screened with bio-layer interferometry (BLI) using an Octet Red 384 platform. The principle and performance of this technique have been described elsewhere [44,45].

**Figure 1:**
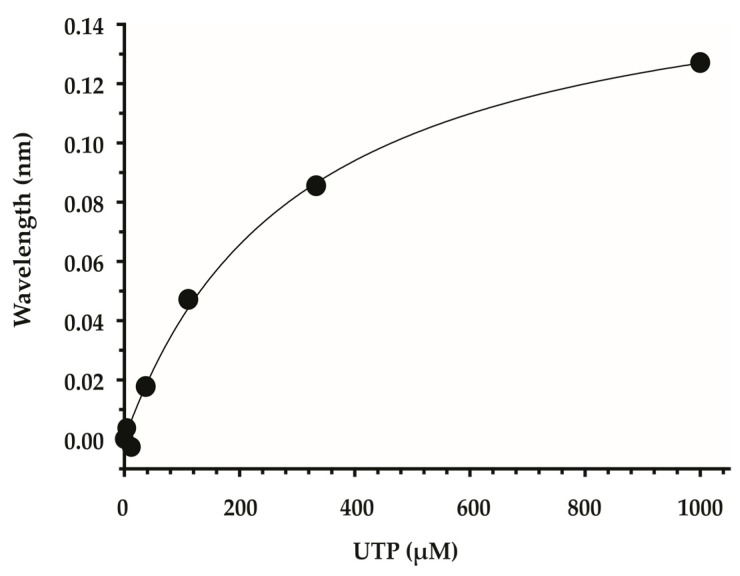
Dose-dependent binding of UTP (in µM) to biotinylated *Leishmania* UDP-sugar pyrophosphorylase (*Lm*USP) immobilized on Super Streptavidin Biosensors.

*Lm*USP was expressed in bacteria with an N-terminal biotin acceptor peptide (BAP epitope), purified, and subsequently biotinylated by incubation with the biotin ligase BirA. Immobilization of biotinylated *Lm*USP to Super Streptavidin (SSA) biosensors was then assessed. Upon assessment by biolayer interferometry, *Lm*USP showed a measured wavelength shift of ~4 nm, with a loading enzyme concentration of 12.5 µg/mL. A natural ligand of *Lm*USP, UTP, was used as the positive control to test for binding. A set of eight SSA biosensors were loaded with a 12.5 µg/mL solution of biotinylated *Lm*USP, and immersed in UTP solutions of various concentrations, ranging from 1.3 µM to 1 mM. Despite the sub-optimal immobilization level of *Lm*USP on the SSA biosensors, UTP bound in a dose-dependent manner (Figure 1). In this experiment, the estimated equilibrium dissociation constant (K_D_) for UTP was 89 µM, which correlates well with the Km value of 100 µM reported previously [21].

**Figure 2:**
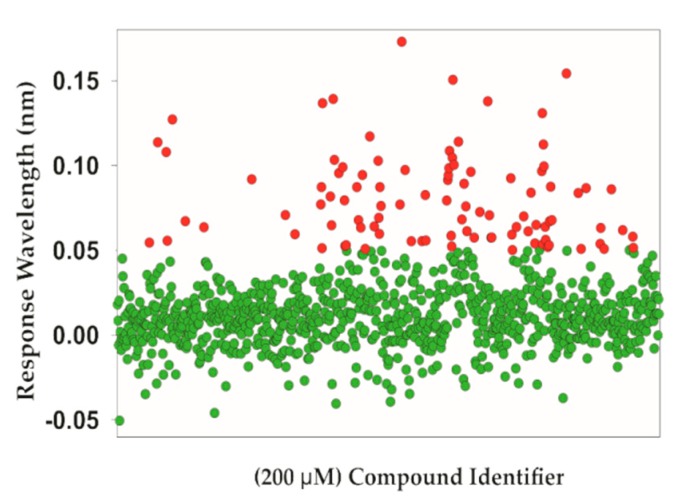
Responses for the bio-layer interferometry screen with *Lm*USP. The binding curve of all fragments was analyzed with ForteBio software and plotted as compounds (*X*-axis) versus response. Fragments with a response > 0.05005, highlighted in red, were selected for a second screen.

The small molecule fragments library was then screened against *Lm*USP to define the binding profile of each fragment. Compounds were initially tested at a concentration of 200 µM, and excess UTP (200 µM) was added to the running buffer in order to keep the enzyme in its physiological UTP bound state. Fragments with a response level greater than 0.05005 (corresponding to a median response of 0.0082 + 3x robust standard deviation of 0.01395) were considered as hits (Figure 2). Based on this equation, 96 fragments, reflecting a primary hit rate of 9.9%, were selected for a follow-up study.

The primary hits were further confirmed by a second screen with a 7-point concentration series from 2 µM to 500 µM. In this validation experiment, 57 fragments showed stoichiometric binding to the target, with approximate binding constants K_D_ ranging from ~60 µM to 480 µM (Supplementary Table S1). Table 1 shows the four fragments that bind to *Lm*USP with an estimated K_D_ value of less than 100 µM. Typically, fragment molecules have a low affinity for the enzyme (K_D_ ≥ 100 µM), but most of the atoms participate directly in the binding process [46]. These fragment molecules can thus provide valuable scaffolds for inhibitor development.

### 2.2. Selecting the First-Generation Inhibitors

**Figure 3:**
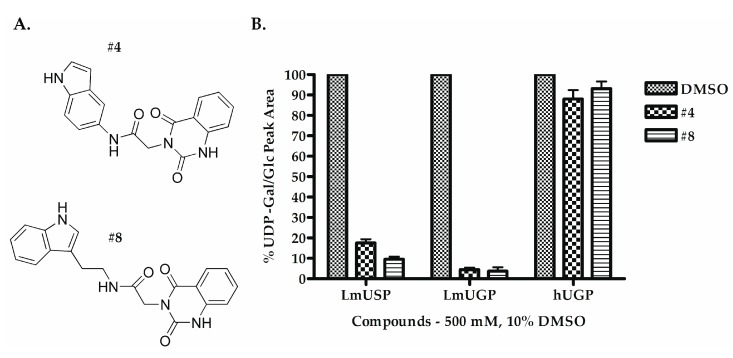
Structure and in vitro inhibitory activity of compounds **#4** and **#8** against *Lm*USP, *Lm* UDP-glucose pyrophosphorylase (UGP), and *h*UGP. (**A**) Structure of compounds **#4** and **#8**. (**B**) In vitro activity of *Lm*USP, *Lm*UGP, and *h*UGP in the absence (DMSO control), and the presence of 500 µM compounds **#4** or **#8**. Compounds were dissolved in DMSO, and all assays were performed in the presence of 10% DMSO. Data were normalized to the DMSO control. Error bars are given as standard deviations from three independent experiments, each with three technical replicates.

To validate the identified scaffolds, we chose fragment DDD00808259, which showed the highest degree of binding in the primary screen, and a low K_D_ value of 67.6 µM, and searched for commercially available compounds containing similar chemical epitopes. Eleven compounds were selected from a structural similarity search with this fragment against the commercially available ChemBridge screening compound database (Supplementary Figure S1). These compounds followed the Lipinski’s rule of five (MW < 500 Da; ClogP < 5; H-bond donors < 5; H-bond acceptors < 10). Their ability to inhibit *Lm*USP in vitro activity was tested by using 500 µM compounds with a final dimethyl sulfoxide (DMSO) concentration of 10%. Compounds **#4** and **#8** (Figure 3A) inhibited UDP-Gal production by at least 80% compared to the DMSO control, which was set to 100% (Figure 3B) whereas the other compounds only moderately inhibited *Lm*USP (less than 30% inhibition). Moreover, at 500 µM, compounds **#4** and **#8** inhibited more than 95% of *Lm*UGP activity (Figure 3B). In contrast, the activity of *h*UGP was decreased by less than 20% (Figure 3B). The compounds thus show selectivity towards the parasite pyrophosphorylases.

**Figure 4:**
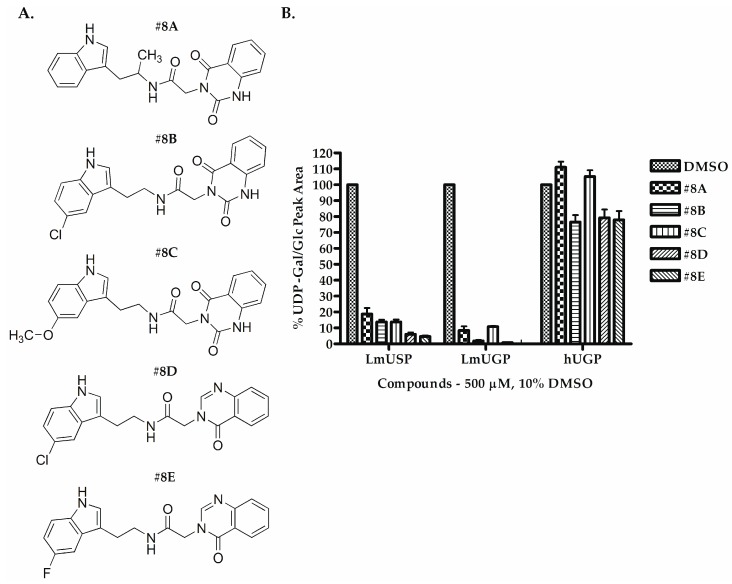
Structure and in vitro inhibitory activity of compounds **#8A** to **#8E** against *Lm*USP, *Lm*UGP, and *h*UGP. (**A**) Structure of compounds **#8A** to **#8E**. (**B**) In vitro activity of *Lm*USP, *Lm*UGP and *h*UGP in the absence and presence of 500 µM compounds **#8A** to **#8E**. Compounds were dissolved in DMSO, and all assays were performed in the presence of 10% DMSO. Data were normalized to the DMSO control. Error bars are given as standard deviations from three independent experiments, each with three technical replicates.

Five commercially available analogs of compound **#8** were subsequently tested for activity against *Lm*USP, *Lm*UGP, and *h*UGP, at an initial concentration of 500 µM. The compounds, named **#8A** to **#8E** (Figure 4A), showed more than 85% reduction of *Lm*USP and *Lm*UGP in vitro activity (Figure 4B). At this high concentration, compounds **#8B**, **#8D** and **#8E** also showed a clear inhibition of *h*UGP (Figure 4B).

The compounds **#4**, **#8** and **#8A** to **#8E** were subsequently used in dose-response studies to measure the corresponding IC_50_ (inhibitor concentration yielding 50% inhibition) values against *Leishmania* USP and UGP. The IC_50_ values based on sigmoidal dose-response curves (presented in Supplementary Figures S2 and S3) are summarized in Table 2.

Compound **#8** was found to have a slightly higher potency toward *Lm*USP than **#4**. Substitution of the indole ring carbon 6 in **#8** by chlorine or a methoxy group resulted in compound **#8B** and **#8C** respectively (Figure 4A). Interestingly, the chlorine substituent increased the inhibitory activity towards both parasite pyrophosphorylases, whereas the more bulky methoxy group decreased it. Similarly, the presence of a methyl group in **#8A** compared to **#8** decreased the potency of the compound. Finally, compounds **#8D** and **#8E** resemble **#8**, but they present a different orientation of the quinazolinone group, and differ from each other by the presence of a chlorine or fluorine substituent at the C6 indole group. Compound **#8E** was identified as the best inhibitor of the parasite pyrophosphorylases, with an IC_50_ value of below 50 µM, for both *Lm*USP and *Lm*UGP.

### 2.3. Predictions of the Compounds’ Binding Sites on LmUGP and LmUSP

**Figure 5:**
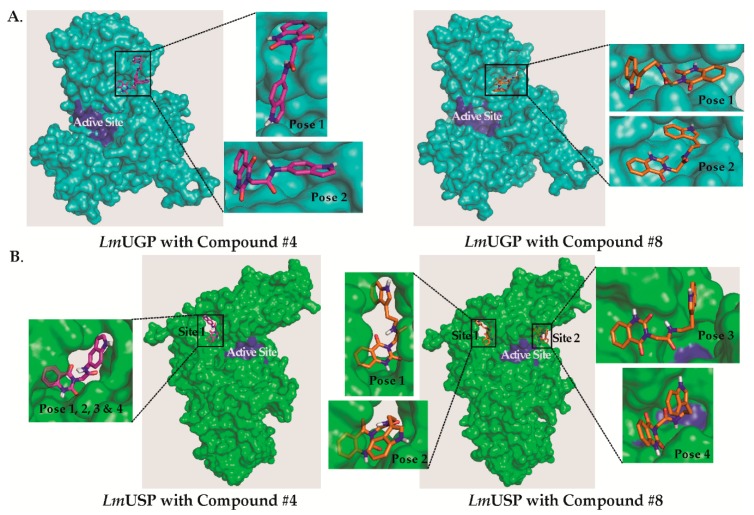
Predicted binding sites of compounds **#4** and **#8** to *Lm*UGP and *Lm*USP. (**A**) Surface representation of *Lm*UGP in cyan, with the active site colored in blue, and compound **#4** (**left**) or compound **#8** (**right**) bound to the allosteric site (**B**) *Lm*USP in green with the active site colored in blue with compound **#4** (**left**) bound to site 1 or compound **#8** (**right**) bound to site 1 and site 2.

Computational docking was used to predict the favored binding sites for compounds **#4** and **#8** on *Lm*UGP and *Lm*USP (Figure 5) using AutoDock software. Both compounds were predicted to bind to *Lm*UGP at an allosteric site that was previously shown to be functionally relevant (Figure 5A) [42]. Inhibitor binding in this area is expected to impede the closing of the *Lm*UGP catalytic pocket, which is necessary for an efficient enzymatic reaction [42,47].

Docking to *Lm*USP suggested that compound **#4** preferentially binds to one site, here named site 1, located in the proximity of the active center of the enzyme (Poses 1, 2, 3, and 4). This site is also predicted to be the preferred binding site of compound **#8** (Poses 1 and 2), but this last compound may also bind to site 2 (Pose 3 & 4) (Figure 5B). Table 3 shows the binding energy corresponding to the four preferred orientations of each compound to *Lm*USP.

Docking of compounds **#4** and **#8**, along with other identified inhibitors (**#8B**, **#8D**, and **#8E**) was repeated with Schrödinger Maestro software. The docking results confirmed the binding sites for compounds **#4** and **#8**. The top binding site of inhibitors **#8B**, **#8D**, and **#8E** was at the same allosteric site of *Lm*UGP and site 1 of *Lm*USP (Supplementary Figures S4 and S5).

### 2.4. Binding Site Validation through Site-Directed Mutagenesis

To analyze if the predicted binding sites have any functional relevance concerning the *Lm*USP activity, site-directed mutation studies were performed. Initially, valine 330 and valine 199, located at sites 1 and 2 respectively, were mutated to a tryptophan residue to introduce steric hindrance, which might block the structural transition associated with protein catalysis. As seen in Table 4, these mutations considerably decreased the affinity of *Lm*USP for its substrate Gal-1P (higher K_m_) and led to lower catalytic efficiencies. Since the V330W mutation resulted in a higher V_max_, we hypothesized that the π-interaction between the introduced tryptophan 330 and phenylalanine 383 (Supplementary Figure S6) might be responsible for a conformation favoring catalysis. We thus mutated F383 to arginine, and also generated an *Lm*USP mutant carrying both the V330W and F383R mutations. Both the single mutation F383R and double mutationV330W/F383R strongly reduced the overall catalytic efficiency of the enzyme. These results demonstrate that site 1 and 2, although they are remote from the active center, are connected to the catalytic activity of LmUSP, and sterically blocking these sites inhibits the enzyme. Site 1 and 2 can therefore be considered as potential allosteric inhibitory sites.

## 3. Discussion

The available treatment options for leishmaniasis are limited to pentavalent antimonials, amphotericin B, miltefosine, paromomycin, sitamaquine, pentamidine, or a combination of these drugs when resistance is reported. Except for sitamaquine, these are wide-spectrum drugs that were not originally developed for treating leishmaniasis [8]. To date, there is no vaccine against leishmaniasis.

Biosynthesis of UDP-Glc and/or UDP-Gal by *Leishmania* promastigotes is essential for parasite growth and survival in vitro [37]. The two enzymes, UGP and USP, found in all *Leishmania* species, are essential for the production of these activated sugars. They contribute to the synthesis of the parasite glycocalyx, and an unusual DNA base J that is likely involved in parasite growth and transcription regulation [17]. Since USP complements the activity of UGP, it would be pivotal to inhibit both enzymes for a clinical outcome. Moreover, the active sites of the human enzyme *h*UGP and parasite pyrophosphorylases show common structural details, so that potential cross-reactivity has to be considered during inhibitor design. We have previously described the detailed enzymatic cycle of *Lm*UGP and we highlighted important structural and mechanistic differences between *Leishmania* and human UGP [34,35,47]. Like *Lm*USP, *Lm*UGP is an active monomer that undergoes large structural changes during its enzymatic cycle. Binding of UTP to the enzyme leads to the formation of the glucose-1-phosphate binding pocket. Binding of this second substrate triggers further conformational changes that stabilize the substrates, and “lock” the enzyme in a compact form that is optimal for the catalytic reaction [47]. In contrast, *h*UGP is active as an octamer, and it uses an intermolecular lock mechanism involving inter-subunit contacts, and it is associated with more restricted molecular movement [34,35]. These mechanistic differences might be exploited to develop selective inhibitors of the parasite enzymes as, recently demonstrated by the identification of an allosteric inhibitor that selectively affects *Lm*UGP [42].

**Figure 6:**
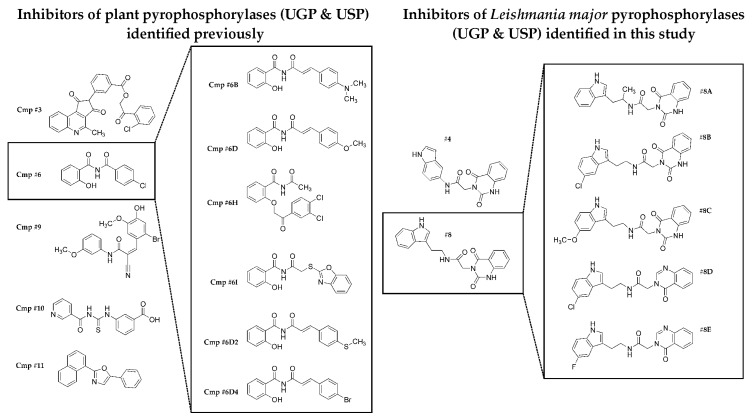
Structural comparison between the *Leishmania major* pyrophosphorylases inhibitors identified in this study, and the previously reported inhibitors of plant pyrophosphorylases [25,51].

Several other studies have aimed at identifying specific inhibitors of pyrophosphorylases to selectively target trypanosomatid parasites. In particular, inhibitors of *Leishmania* GDP-mannose pyrophosphorylase and *T. brucei* UDP-*N*-acetylglucosamine pyrophosphorylase, two genetically validated targets, have been reported, and they display selectivity over their human orthologues [48,49,50]. Some of these inhibitors show anti-parasite activity. Additionally, inhibitors of both UGP and USP have been developed, to study the importance of UDP-sugars in plants (Figure 6) [24,25,51]. For this purpose, the potential inhibition of human UGP was not a concern, and it was thus not tested. Since the compounds inhibited both UGP and USP, the authors have suggested that they target the active site of the enzymes. With the aim of identifying inhibitors that are selective over human UGP, we performed a small molecule fragment screen with *Leishmania major* USP as a target. This screen identified 57 small molecules binding to *Lm*USP, with an estimated K_D_ ranging from ~ 60 µM to 480 µM. Because of their small sizes, the potency of such molecules is rather low, but most of the atoms contribute directly to the binding process [46]. The identified fragments are dissimilar from the substrates of the enzyme, and are thus valuable scaffolds for the development of allosteric inhibitors. To validate the result of this screen, we purchased commercially available compounds containing epitopes that are identical or similar to the most potent fragment, and tested for inhibitory activity against *Lm*USP in vitro. Two of the 11 compounds initially chosen showed clear inhibition of *Lm*USP activity with IC_50_ in the 100 µM range (compounds **#4** and **#8**). Remarkably, these two compounds also inhibit *Lm*UGP activity, but they have little effect on the human enzyme. Five derivatives of compound **#8** were also analyzed, and this led to the identification of an inhibitor of both parasite pyrophosphorylases, with IC_50_ values < 50 µM (compound **#8E**). These analogs of compound **#8** are also selective for the parasite enzymes, but a modest inhibition of *h*UGP is observed at high compound concentration. The inhibitors identified in this study differ from the previously described inhibitors of USP (Figure 6). They include a quinazolinone, as the fragment selected from the library screen, and an indole group.

Computational methods were used to explore possible binding modes of the inhibitors to *Lm*UGP and *Lm*USP. Interestingly, the preferred predicted binding site of the inhibitors to *Lm*UGP was the previously reported allosteric site [42]. In the case of *Lm*USP, two different cavities located outside, but in the proximity of the catalytic site, were identified as preferential binding sites of the inhibitors. Point mutations of residues present at these sites resulted in a drastic reduction of *Lm*USP catalytic efficiency, demonstrating the potential for targeting these cavities for allosteric regulation. The bound compounds might be inhibiting *Leishmania* enzymes by hindering the structural transition that is associated with enzymatic activity [47]. Unfortunately, because of the limited solubility of the identified inhibitors, we did not succeed in obtaining a crystal structure of *Lm*USP or *Lm*UGP with the bound inhibitors. Such structures could shed light on the exact binding modes and epitopes that are making contact with protein residues to assist ligand optimization, for improved binding and selective inhibition. Chemical derivatization of the compounds, and structure–activity relationship (SAR) studies might improve their properties with regard to solubility, binding affinity, and specificity.

In conclusion, this study describes the identification of first-generation inhibitors of *Leishmania major* USP and UGP, based on small-molecule fragment library screening. The identified inhibitors were predicted to bind to allosteric pockets of *Lm*USP and *Lm*UGP that have been validated by mutagenesis experiments, and they display some specificity over human UGP. These compounds that follow the Lipinski’s rule of five, as well as the structurally diverse molecular scaffolds that were identified in the library screening, are promising starting points for the development of potent and selective inhibitors of *Leishmania major* USP.

## 4. Materials and Methods

### 4.1. A BLI Screen Using Recombinant LmUSP

A vector containing the His(6)-biotin acceptor peptide (BAP) tag was kindly provided by Prof. Mike Ferguson from the University of Dundee. The BAP tag was inserted in the *Xba*I and *Nde*I restrictions site of the previously described pET22b-*Lm*USP [21]. The plasmid with insert was then transformed into the *E. coli* BL21(DE3) expression strain, and *Lm*USP was expressed and purified, as described previously [21]. Exactly 2 mg of TEV protease was added to cleave off the His tag. The protein was dialyzed in 3 × 1 L Buffer C (50 mM TRIS pH 8.0, 150 mM NaCl, 10 mM MgCl_2_). A concentration of 100 µM d-Biotin, 500 µM ATP, and 200 µM BirA enzyme were added to the protein and incubated for 18 hr for biotinylation. The sample was concentrated to 11 mL, using a Vivaspin 20 30 kDa PES centrifugal concentrator (Sartorious, Stonehouse, United Kingdom). The protein was loaded onto a Superdex 200 XK26/60 equilibrated with Buffer C on the AKTA Pure at 4 °C. The eluted monomeric protein was then concentrated to 0.98 mg/mL using a Vivaspin 20 30 kDa PES centrifugal concentrator, and used for loading onto super streptavidin (SSA) biosensors (Fortebio, Shanghai, China, Part No. 18-5057, Lot No. 1612162).

The loading response to SSA biosensors was measured to find the optimal protein concentration to be used for the screen. Briefly, biosensor tips were hydrated in assay buffer (50 mM Tris pH 8.0, 150 mM NaCl, 10 mM MgCl_2_) for a minimum of 5 min before being sequentially transferred to assay wells containing 1) assay buffer (60 s) to establish the baseline, 2) protein at 12.5, 25, or 50 µg/mL (900 s) to measure protein loading, and 3) assay buffer to assess the reversibility of protein binding to the sensor. The concentration that was optimal for screening was identified to be 12.5 µg/mL. A concentration-effect curve for UTP (1.4 µM to 1 mM) was carried out, using SSA sensors with 12.5 µg/mL immobilized *Lm*USP, to derive an affinity estimate (Kd).

For fragment screening, 16 SSA biosensors were prepared by loading *Lm*USP (12.5 µg/mL) for 900 s; free streptavidin sites were then blocked with biocytin (10 µg/mL) for 120 s, and finally washed in buffer W (50 mM Tris pH 8.0, 150 mM NaCl, 10 mM MgCl_2_, 200 µM UTP) for 60 s. In parallel, another 16 SSA sensors were loaded with 10 µg/mL biocytin alone, which acts as a quenching agent to block streptavidin sites on the sensor surface, and then washed with buffer W. These were used as reference sensors.

Initial screening was performed with DDU fragment set (965 fragments) at a single point concentration of 200 µM. The assay consisted of a 60 s baseline step, a 60 s association step, and a 60 s dissociation step. The assay was repeated with the streptavidin blocked control biosensors. Response levels for each compound were calculated by baseline subtraction, and the subtraction of the reference responses from the *Lm*USP responses. From the initial putative hits, compounds were further validated in a 7-point concentration series using 3-fold dilutions of each fragment ranging from 500 µM to 2.05 µM. Data were processed, and kinetic parameters were calculated, using the ForteBio software. A visual inspection of curves alongside steady state fitting was used to identify fragments of interest.

### 4.2. Fragment Similarity Search

The structure of the top fragment was drawn in the Ketcher platform of the ChemBridge (ChemBridge Corporation, San Diego, CA, USA) chemical store website Hit2Lead (https://www.hit2lead.com/) [52], and a similarity-based search was performed against the screening compounds database. The search result was visually inspected to select 11 compounds with a similar chemical epitope: **#1** (CID 9245967), **#2** (CID 9214059), **#3** (CID 9252929), **#4** (CID 9207718), **#5** (CID 9231249), **#6** (CID 9270353), **#7** (CID 9195974), **#8** (CID 9206277), **#9** (CID 9280745), **#10** (CID 9329111) and **#11** (CID 9212920). These were purchased from the ChemBridge Hit2Lead store to test for inhibition against *Lm*USP, *Lm*UGP, and *h*UGP. Five structural analogues of the initial hit compound **#8**: **#8A** (CID 9219493), **#8B** (CID 9277739), **#8C** (CID 9214406), **#8D** (CID 9251350), and **#8E** (CID 9259570) were also purchased and tested for inhibition. The compounds were dissolved in 100% DMSO, and stored in glass vials at a concentration of 100 mM in the dark.

### 4.3. Protein Purification and Mutant Generation

*Lm*USP, *Lm*UGP, and *h*UGP proteins were produced and purified as described previously [21,26,35]. Q5 Site-Directed Mutagenesis Kit from New England BioLabs was used to generate different *Lm*USP mutants. The primer pairs GGAGTACAACTGGTTTGCCGAGGTCTCGCGC and ACGTTCGCGACCAGCCAC were used for the generation of the *Lm*USP V330W mutant, GTGCCGGAGCGCATCAATCCCAAG and GATACCGTGCGACTCCCGCAG for the *Lm*USP F383R mutant, and GCAGGGGCAGTGGTTCTGTTTTGC and TTGAGCACATGCAAGTTG for the *Lm*USP V199W mutant. Mutations were confirmed by sequencing of the plasmids.

### 4.4. Enzyme Activity Assay and Dose Response Study

Compounds were tested at a concentration of 500 µM with 20 nM enzyme in an high-performance liquid chromatography (HPLC)-based assay at a final reaction volume of 100 µL. The DMSO concentration in the reaction was kept at 10%. The reaction buffer consisted of 10 mM Tris-HCl pH 8.0, 2 mM MgCl_2_, 10 mM NaCl, 0.005 U of inorganic pyrophosphatase (Thermo Fisher Scientific, Vilnius, Lithuania), and 1 mM Dithiothreitol (DTT) (for *Lm*USP and *Lm*UGP only). Protein was incubated with the inhibitors and UTP (final concentration of 0.8 mM in reaction) for 30 min at room temperature, before the addition of the reaction buffer. The reaction was started with the addition of Gal-1P for *Lm*USP and Glc-1P for *Lm*UGP and *h*UGP (final reaction concentration of 2 mM) and incubated for 30 min at 25 °C, before heat inactivation at 95 °C for 15 min. The samples were spun down and the supernatants applied to a Dionex CarboPac™ PA10 column (Thermo Fisher Scientific, Sunnyvale, CA, USA). UDP-Gal or UDP-Glc (products) were separated from UTP (substrate), UMP, and UDP (both, UTP hydrolysis products) with a NaCl gradient elution ranging from 0 to 500 mM NaCl over 13 min at 0.6 mL/min. Absorption of the uracil moiety was detected at 260 nm. The peak area of UDP-Gal or UDP-Glc in reactions with inhibitors was compared to a control containing 10% DMSO. Protein concentrations were calculated from the measured UV-absorbance at 280 nm (Implen NanoPhotometer N60, Implen GmbH, München, Germany), and the proteins’ individual extinction coefficients were calculated using the online ProtParam tool (http://web.expasy.org/protparam/) [53]. For the dose-response study and IC_50_ determination, the same procedure was performed with the inhibitor concentrations ranging from 7 µM to 500 µM. Corresponding IC_50_ values were calculated by using nonlinear regression analysis with variable slope, in GraphPad Prism version 4.00 (GraphPad Software, San Diego, CA, USA). 

### 4.5. Docking Analysis

Computational docking experiments with AutoDock MGL tools 1.5.6 [54,55] and Schrödinger Maestro 2018-4 (Schrödinger, LLC, New York, NY, USA, 2018) were performed to identify the compound binding sites on the protein. UTP- and UTP analog bound-structures of *Lm*USP and *Lm*UGP (PDB ID: 3OH0, 4M28), respectively, were used to perform the docking experiments. For docking with AutoDock, crystallization water was retained in the protein structure, and all other hetero molecules were removed before docking. Protein and compound structures were then prepared in AutoDock, and a grid area of 126 × 126 × 126 points, covering protein surface inclusive of the active site, was assigned using AutoGrid, with grid spacing set at 0.4 Å. A Lamarckian genetic algorithm [55] was implemented to dock the compounds with software settings: the population size of 150, the maximum number of energy evaluations set to 2,500,000, the maximum number of generations set to 27,000, and the number of genetic algorithm runs set to 100 [56].

For docking with Schrödinger Maestro, pre-docking minimization and optimization of the ligand structures were performed with the ligprep module, using an OPLS_2005 force-field. Protein PDB structures were prepared in a protein preparation wizard. Crystallization water molecules making at least two hydrogen bonds with protein atoms were retained, and all other hetero-atoms were removed from the receptor structures before the docking procedure. Grid maps were generated for both the proteins, covering protein surface, including the active site. The Glide extra-precision (XP) docking procedure was followed for docking the ligands to the receptors [57]. The resulting top binding poses were exported to PyMOL (The PyMOL Molecular Graphics System, Version 2.0 Schrödinger, LLC, New York, NY, USA) for visualization and image creation.

### 4.6. Enzyme Kinetics

Michaelis–Menten kinetic parameters were determined for the *Lm*USP wild-type (WT) and its mutants, through varying the concentration of Gal-1P at a saturating UTP concentration. Reaction rates were determined using a commercially available EnzChek^TM^ Pyrophosphate Assay Kit (Molecular Probes/Life Technologies Corporation, Eugene, OR, USA) which continuously detects the formation of pyrophosphate during the forward reaction. The assay reaction was run at 25 °C in a 100 μL volume containing 10 mM Tris-HCl pH 8.0, 2 mM MgCl_2_, 0.12 U inorganic pyrophosphatase, 2 U purine nucleoside phosphorylase, 200 µM MESG, 1 mM DTT, 0.8 mM UTP, and varying concentrations of Gal-1P. The enzymatic reactions were carried out in 96-well half-area flat-bottomed microplates (Greiner Bio-One GmbH, Frickenhausen, Germany), and initiated by the addition of UTP with thorough mixing. The formation of products was continuously monitored at 360 nm, using a 96-well microplate reader (Power-Wave^TM^ 340 KC4 system, BioTek^®^ Instruments GmbH, Bad Friedrichshall, Germany). Initial rates were plotted against Gal-1P concentration, and K_m_ and V_max_ values were calculated from the Michaelis–Menten equation, using nonlinear regression analysis with GraphPad Prism version 4.00 (GraphPad Software, San Diego, CA, USA).

## Figures and Tables

**Table 1:** Chemical structure, binding response, and binding affinity of the top four fragment hits from a dose-response binding study with *Lm*USP.

Fragment	Structure	Primary Binding Response	Estimated KD (µM)
DDD00095351	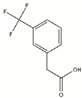	0.127	83.3 µM
DDD00102262	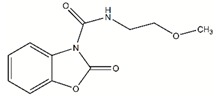	0.077	72.6 µM
DDD00808259	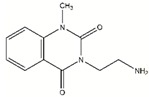	0.1728	67.6 µM
DDD01305586	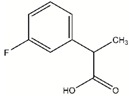	0.1541	60.1 µM

**Table 2:** IC_50_ values of compounds against *Leishmania* USP and UGP at 95% confidence intervals. Compounds were tested at several concentrations from 7 µM to 500 µM.

Compounds	IC_50_ (µM)	
	*Lm*USP (UDP-Gal)	*Lm*UGP (UDP-Glc)
**#4**	135.00–175.00	126.30–134.00
**#8**	78.06–106.60	119.80–151.20
**#8A**	>250	>250
**#8B**	47.17–66.43	55.06–69.13
**#8C**	>250	>250
**#8D**	169.80–194.80	192.70–242.10
**#8E**	40.39–46.29	37.99–46.76

**Table 3:** Calculated ΔG binding energy values (kcal/mol) for the preferred binding orientations of compound **#4** and **#8** to *Lm*UGP and *Lm*USP proteins.

Ligand Pose	Compound #4 Binding Energy (kcal/mol)		Compound #8 Binding Energy (kcal/mol)	
	*Lm*UGP	*Lm*USP	*Lm*UGP	*Lm*USP
Pose 1	−7.61	−7.83	−7.92	−8.22
Pose 2	−7.61	−7.78	−7.71	−7.80
Pose 3	-	−7.74	-	−7.41
Pose 4	-	−7.70	-	−7.30

**Table 4:** Kinetic parameters for Gal-1P with wild-type *Lm*USP and various mutants. The efficiency obtained for the wild-type protein was set to 100%. Errors are given as standard deviations from a single experiment with duplicates.

*Lm*USP	V_max_ (µmol min^−1^ mg^−1^)	K_m_ (µM) Gal-1P	k_cat_ (s^−1^)	k_cat_/K_m_ (µM^−1^ s^−1^)	% Catalytic Efficiency
Wild type	184.4 ± 6.36	529.20 ± 24.60	216.45 ± 7.47	0.409 ± 0.03	100 ± 8.094
V330W	271.70 ± 19.37	1255.00 ± 145.66	319.32 ± 22.77	0.255 ± 0.01	62.25 ± 2.79
F383R	14.77 ± 0.24	364.00 ± 22.20	17.33 ± 0.28	0.047 ± 0.002	11.64 ± 0.52
V330W/F383R	21.76 ± 1.26	276.45 ± 51.26	25.58 ± 1.48	0.093 ± 0.01	22.85 ± 2.92
V199W	76.79 ±2.94	1007.25 ± 18.03	90.25 ± 3.46	0.089 ± 0.001	21.86 ± 0.44

## Supplementary Materials

The following are available online, Figure S1: Scaffold and compounds selected by similarity-based search in the ChemBridge screening compounds database, Figure S2: Nonlinear regression—sigmoidal dose-response curve for the IC_50_ determination of compounds **#4**, **#8**, **#8B**, **#8D** and **#8E** bound to *Lm*USP, Figure S3: Nonlinear regression—sigmoidal dose-response curve for the IC_50_ determination of compounds **#4**, **#8**, **#8B**, **#8D** and **#8E** bound to *Lm*UGP, Figure S4: Surface representation of *Lm*UGP in cyanwith the active site colored in blue, Figure S5: Surface representation of *Lm*USP in green with the active site colored in blue, Figure S6: Phenylalanine 383 (Wild type) and Tryptophan 330 (introduced by point mutation) are hypothesized to favor catalytically active conformation via π- interaction, Table S1: Structure and K_D_ values of all the hits from the BLI-based fragment binding study.
