# Supplementary material for: Identification of Leishmania major UDP-Sugar Pyrophosphorylase Inhibitors Using Biosensor-Based Small Molecule Fragment Library Screening
Source: Molecules. 2019 Mar 12;24(5):996. doi: 10.3390/molecules24050996 (PMC6429087; doi:10.3390/molecules24050996)
Supplement: Supplementary file 1 [file molecules-24-00996-s001.pdf]

**Supplementary Materials:**

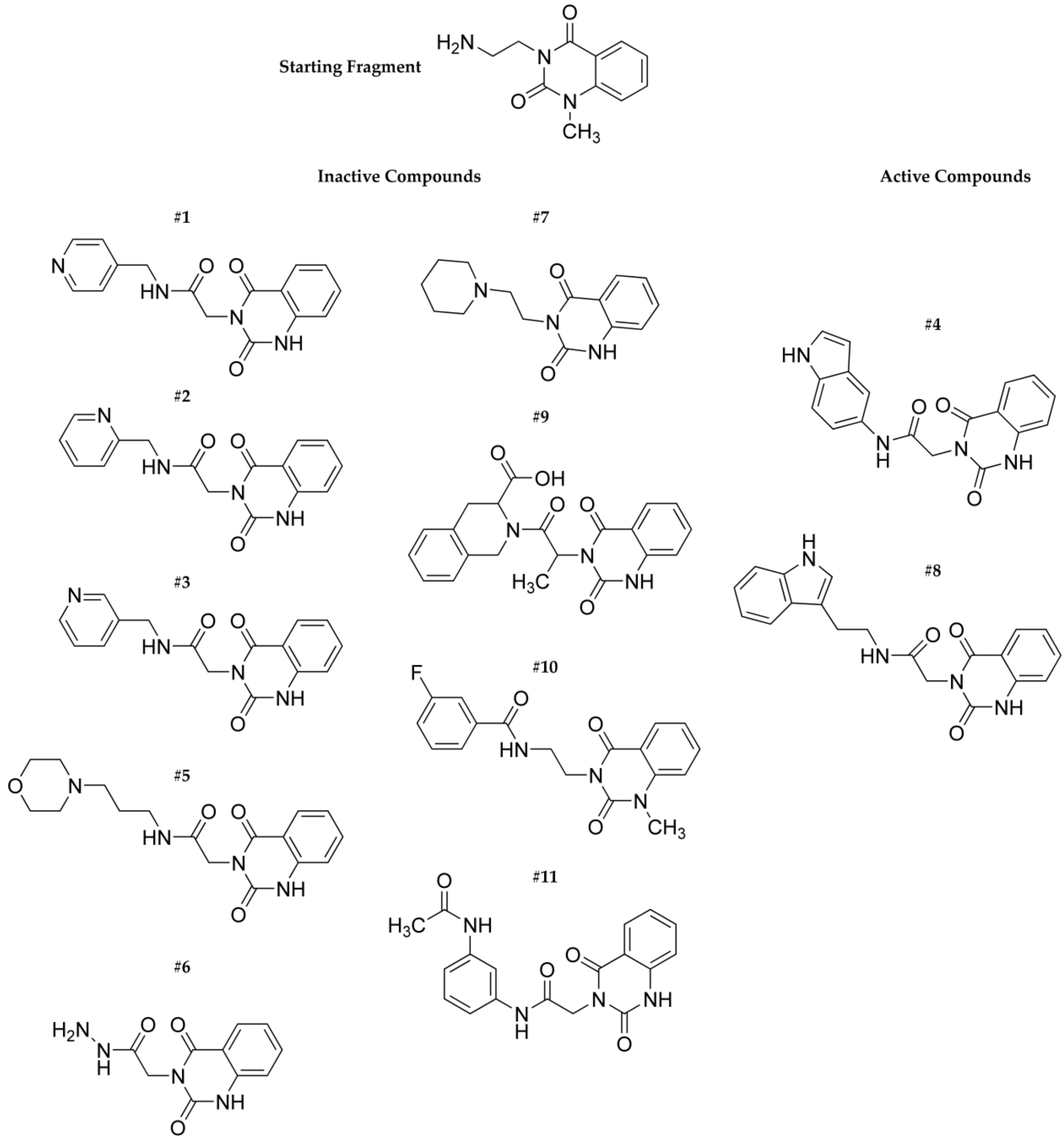


**Figure S1.** Scaffold and compounds selected by similarity-based search in the ChemBridge screening compounds database. Compounds were classified as active if they showed significant inhibition of *Lm*USP activity at 500 μM. The compounds can be identified by their respective ChemBridge ID numbers: #**1** (CID 9245967), #**2** (CID 9214059), #**3** (CID 9252929), #**4** (CID 9207718), #**5** (CID 9231249), #**6** (CID 9270353), #**7** (CID 9195974), #**8** (CID 9206277), #**9** (CID 9280745), #**10** (CID 9329111), #**11** (CID 9212920).

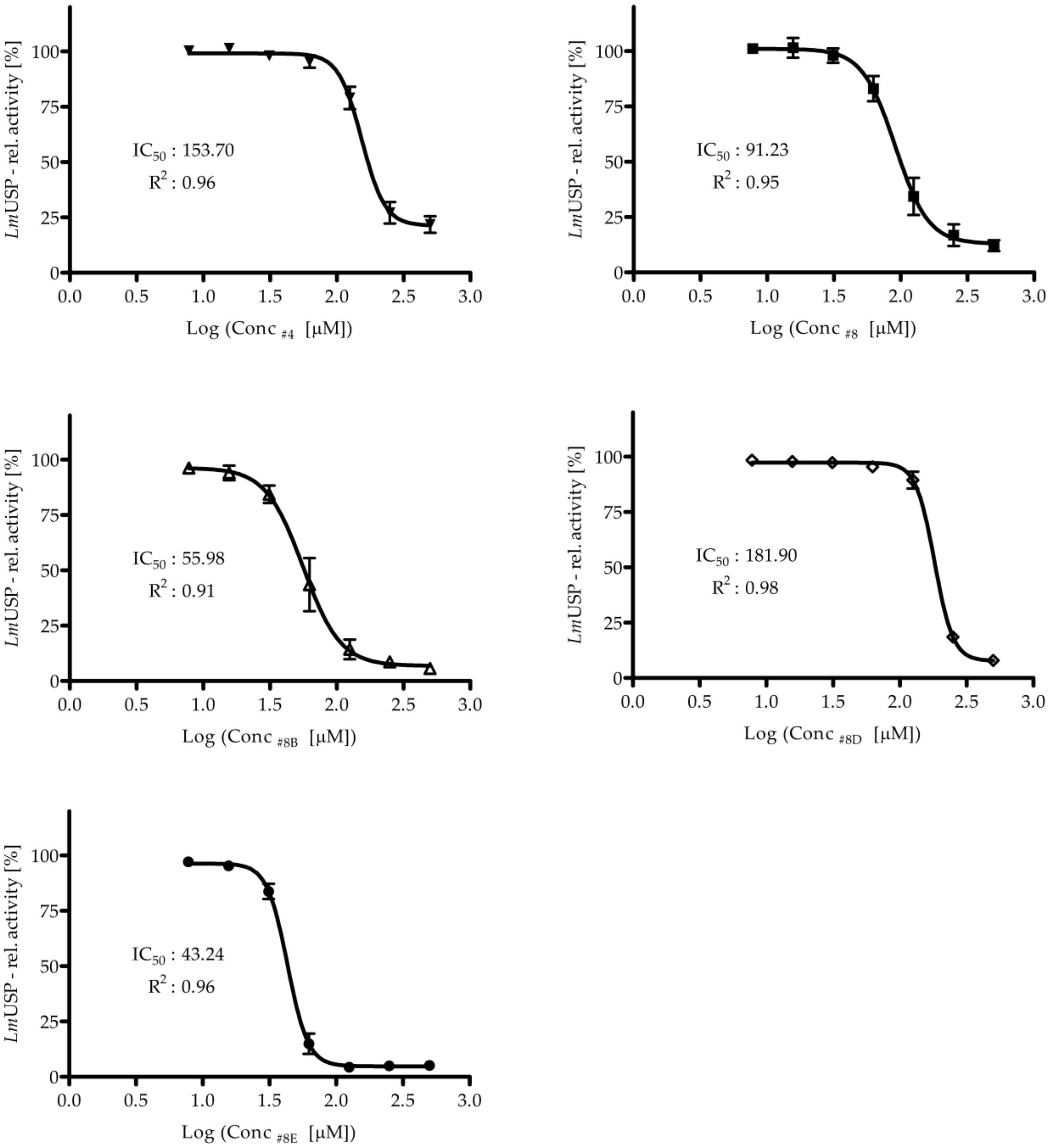


**Figure S2**. Nonlinear regression – sigmoidal dose-response curve for $IC_{50}$ determination of compounds #**4**, #**8**, #**8B**, #**8D** and #**8E** bound to *Lm*USP. Error bars are given as standard deviations from at least 2 independent experiments.

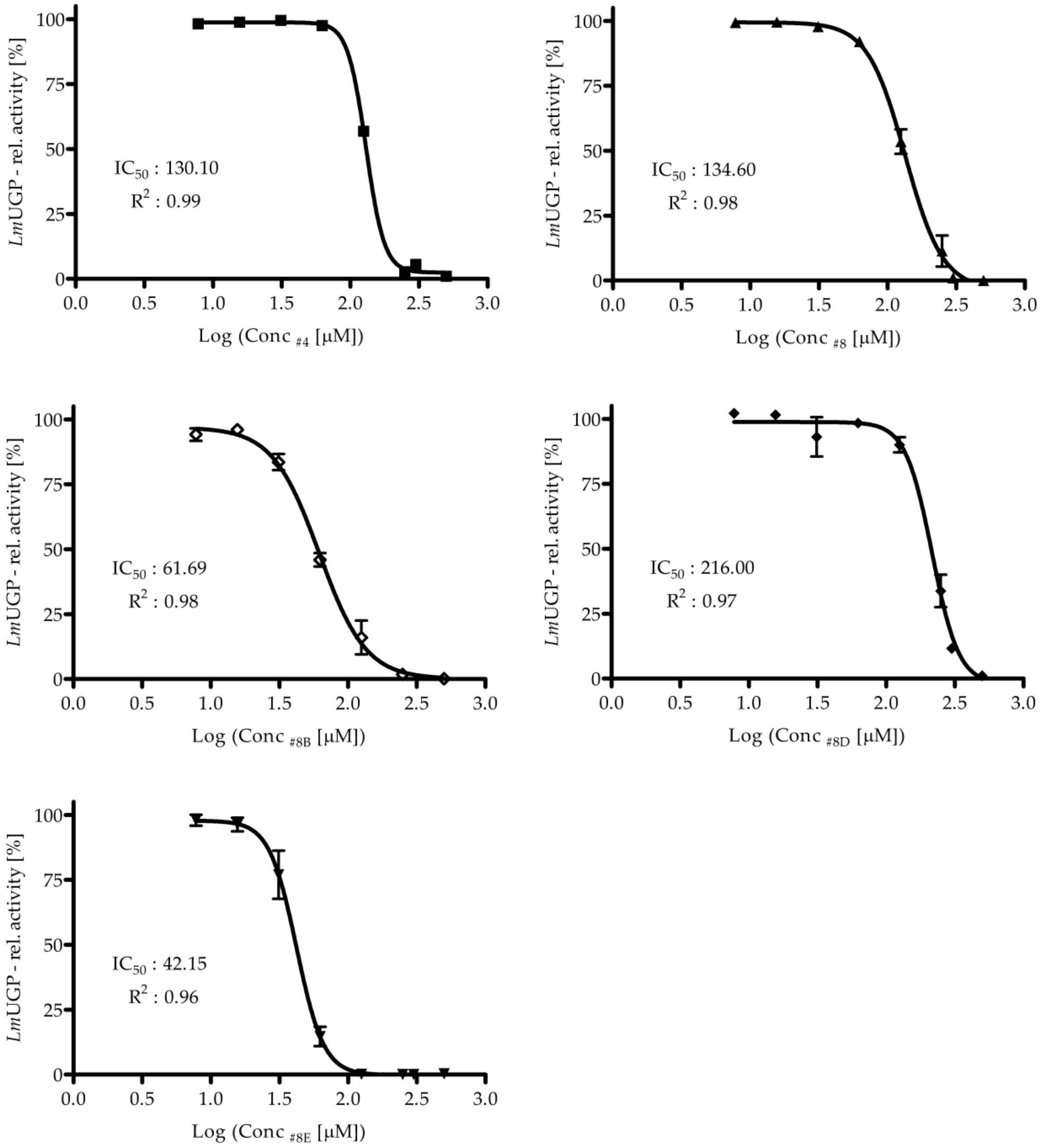


**Figure S3**. Nonlinear regression – sigmoidal dose-response curve for $IC_{50}$ determination of compounds #**4**, #**8**, #**8B**, #**8D** and #**8E** bound to *Lm*UGP. Error bars are given as standard deviations from at least 2 independent experiments.

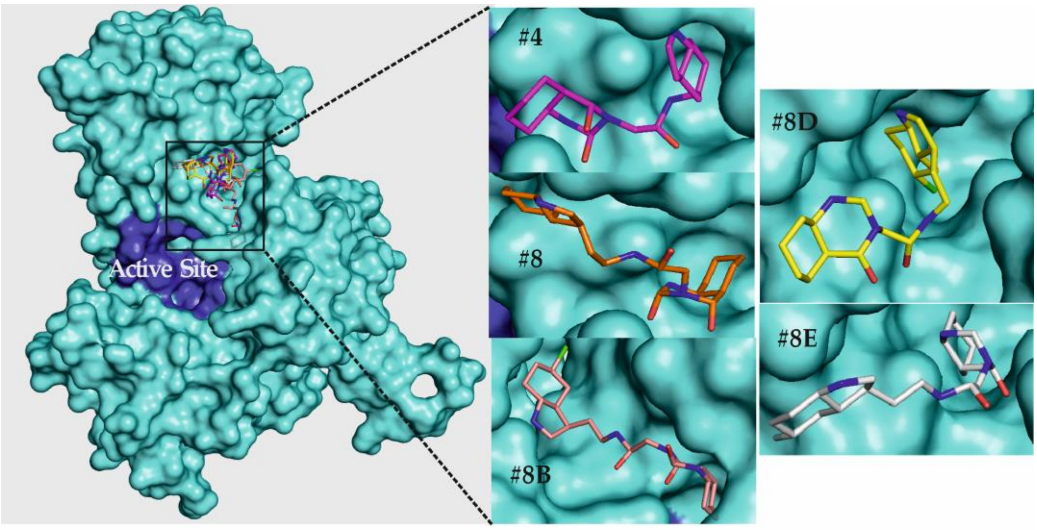


| Compound | GLIDE –XP Docking Score (kcal/mol) |
|---|---|
| **#4** | -9.647 |
| **#8** | -8.186 |
| **#8B** | -10.638 |
| **#8D** | -7.428 |
| **#8E** | -10.099 |

**Figure S4.** Surface representation of *Lm*UGP in cyan with the active site colored in blue. The docked ligands (#**4**, #**8**, #**8B**, #**8D**, and #**8E**) at the allosteric site are shown in stick representation. The table shows the corresponding docking scores.

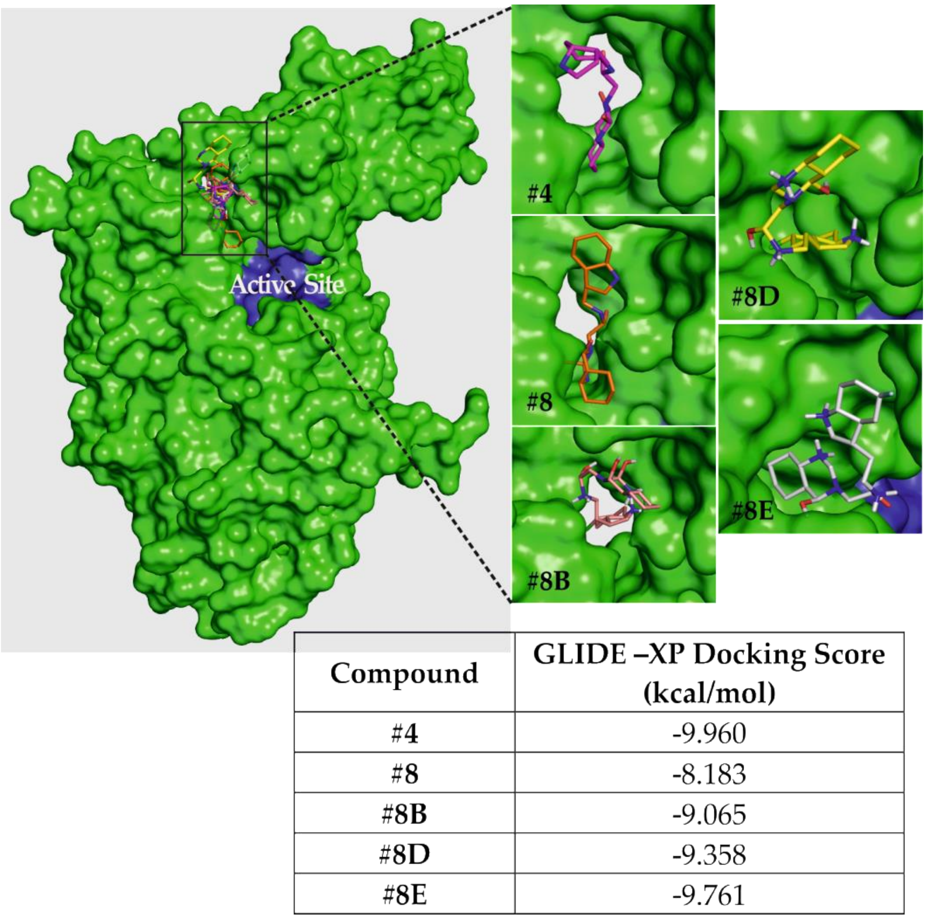


| Compound | GLIDE –XP Docking Score (kcal/mol) |
|---|---|
| **#4** | -9.960 |
| **#8** | -8.183 |
| **#8B** | -9.065 |
| **#8D** | -9.358 |
| **#8E** | -9.761 |

**Figure S5.** Surface representation of *Lm*USP in green with the active site colored in blue. The docked ligands (#**4**, #**8**, #**8B**, #**8D,** and #**8E**) at site 1 are shown in stick representation. The table shows the corresponding docking scores.

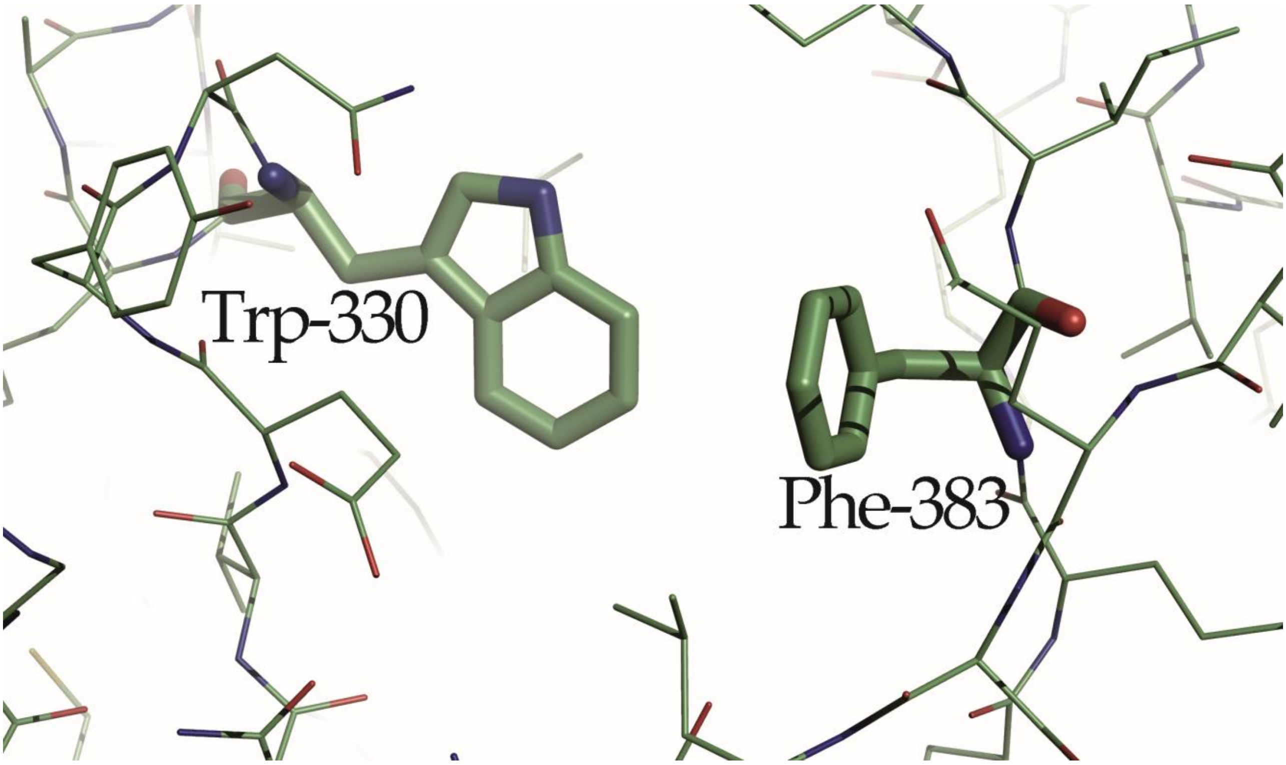


**Figure S6.** Phenylalanine 383 (Wild type) and Tryptophan 330 (introduced by point mutation) hypothesized to favor catalytically active conformation via π- interaction. Mutation was introduced using the 'Mutagenesis' feature in Pymol.

**Table S1.** Structure and estimated $K_D$ values of hits from the BLI based fragment binding study.

| DDU Fragment ID | Structure | Estimated $K_D$ (μM) |
|---|---|---|
| DDD01305586 | 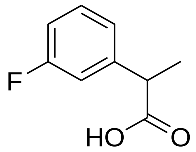 | 60.1 |
| DDD00808259 | 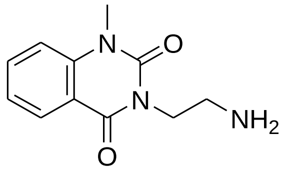 | 67.6 |
| DDD00102262 | 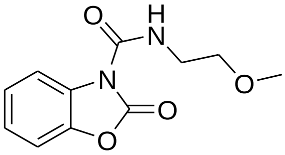 | 72.6 |
| DDD00095351 | 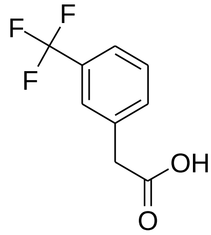 | 83.3 |
| DDD00957472 | 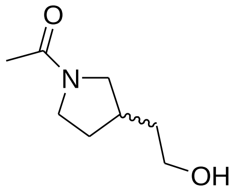 | 105 |
| DDD00957473 | 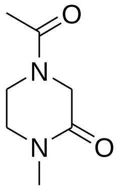 | 120 |
| DDD01305716 | 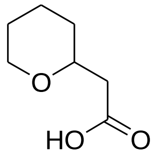 | 120 |
| DDD01085540 | 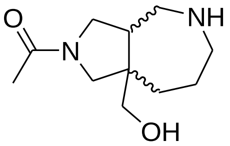 | 135 |
| DDD01085543 | 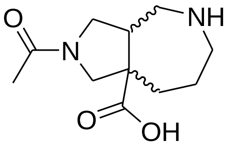 | 137 |

| | | |
|---|---|---|
| DDD01008789 | 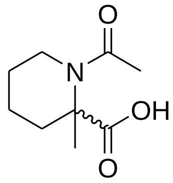 | 140 |
| DDD00805740 | 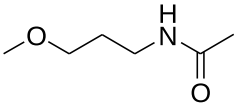 | 156 |
| DDD00957469 | 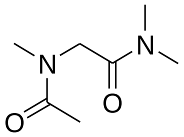 | 156 |
| DDD01085499 | 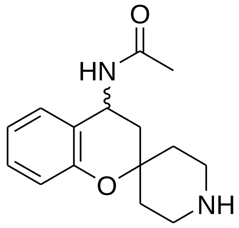 | 156 |
| DDD01085562 | 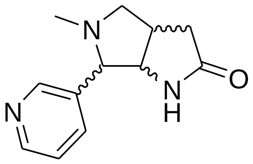 | 157 |
| DDD01012352 | 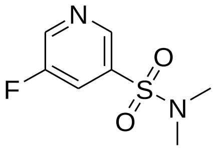 | 161 |
| DDD00771428 | 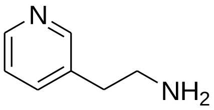 | 163 |
| DDD01085542 | 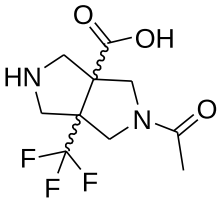 | 167 |
| DDD00103423 | 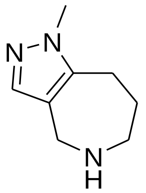 | 173 |
| DDD01008838 | 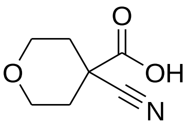 | 173 |
| DDD01008805 | 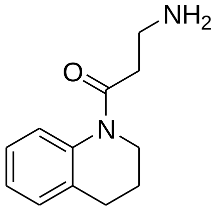 | 176 |

| | | |
|---|---|---|
| DDD00770978 | 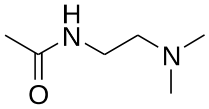 | 181 |
| DDD01008822 | 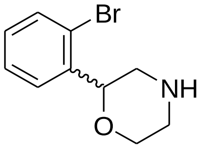 | 185 |
| DDD01269650 | 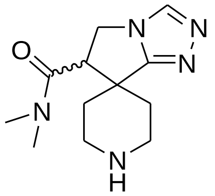 | 194 |
| DDD00805744 | 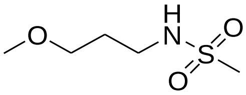 | 204 |
| DDD01269623 | 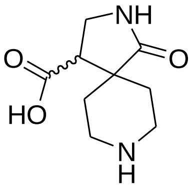 | 207 |
| DDD00771029 | 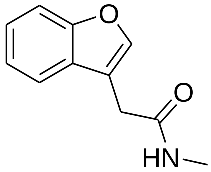 | 210 |
| DDD00103338 | 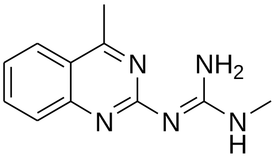 | 212 |
| DDD00957468 | 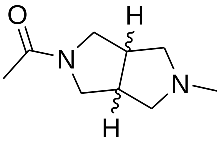 | 220 |
| DDD01269624 | 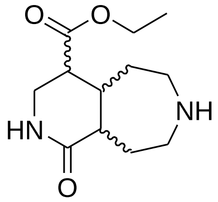 | 220 |
| DDD01008885 | 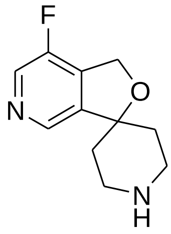 | 221 |
| DDD01008890 | 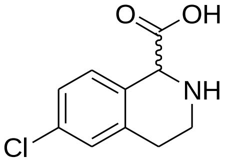 | 223 |

| | | |
|---|---|---|
| DDD00808270 | 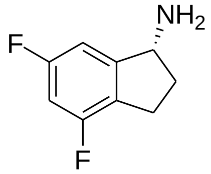 | 247 |
| DDD00203536 | 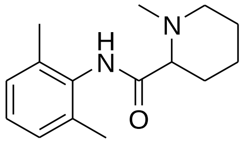 | 247 |
| DDD00121991 | 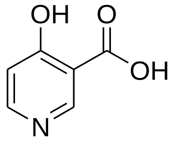 | 250 |
| DDD00327080 | 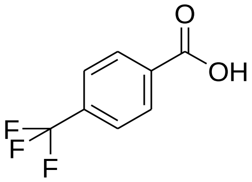 | 251 |
| DDD01085541 | 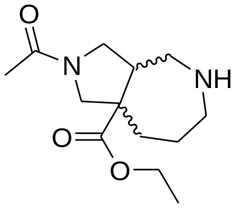 | 251 |
| DDD01305698 | 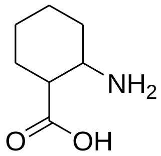 | 256 |
| DDD01008830 | 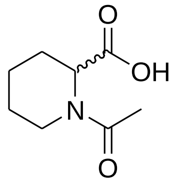 | 264 |
| DDD00771485 | 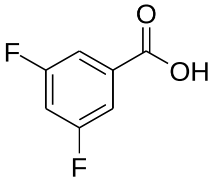 | 267 |
| DDD01085545 | 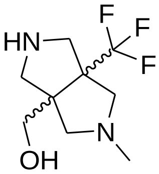 | 270 |
| DDD01008781 | 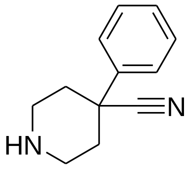 | 274 |

| | | |
|---|---|---|
| DDD01008895 | 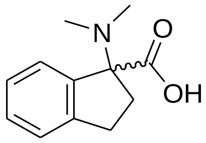 | 283 |
| DDD00923236 | 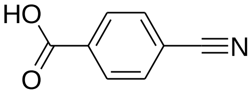 | 286 |
| DDD01085465 | 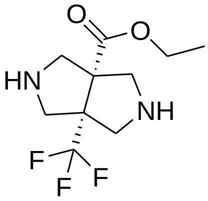 | 295 |
| DDD00321558 | 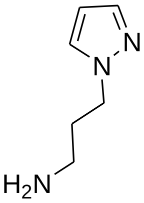 | 296 |
| DDD01024717 | 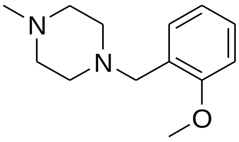 | 297 |
| DDD01305646 | 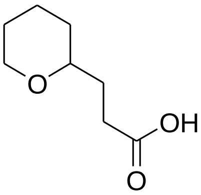 | 318 |
| DDD00362117 | 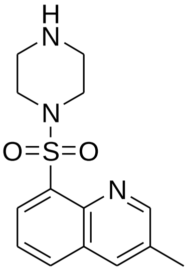 | 320 |
| DDD01085520 | 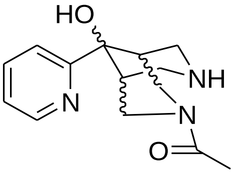 | 328 |
| DDD01008813 | 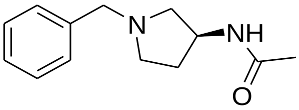 | 355 |
| DDD01305629 | 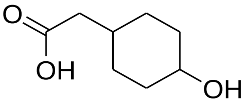 | 388 |
| DDD01305746 | 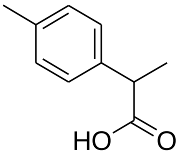 | 391 |

| | | |
|---|---|---|
| DDD01085515 | 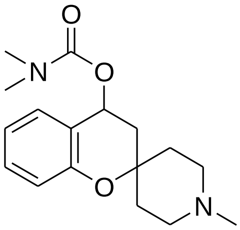 | 395 |
| DDD00910832 | 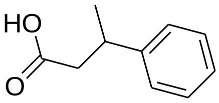 | 432 |
| DDD01512351 | 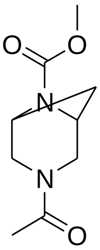 | 452 |
| DDD01305686 | 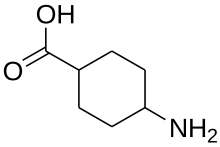 | 457 |
| DDD01512353 | 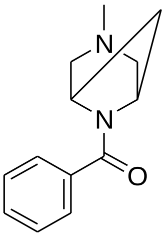 | 481 |
